# Development of gold Immunochromatographic assay strip based on specific polyclonal antibodies against capsid protein for rapid detection of porcine circovirus 2 in Zhejiang province, China

**DOI:** 10.1186/s12917-022-03471-6

**Published:** 2022-10-18

**Authors:** Haojie Ding, Yu Shen, Yafan Gao, Songrui Wu, ChengZuo Xie, Hao Sun, Hongli Zhang, Hongchao Sun, Ying Shan, Jianzu Ding, Bin Zheng, Shaohong Lu, Xunhui Zhuo

**Affiliations:** 1 School of Basic Medical Sciences and Forensic Medicine, Hangzhou Medical College, Hangzhou, Zhejiang China; 2 Zhejiang Center of Animal Disease Control, Hangzhou, Zhejiang China; 3 Department of Animal Parasitology, Institute of Animal Husbandry and Veterinary Medicine, Zhejiang Academy of Agricultural Science, Hangzhou, Zhejiang China; 4 Department of Veterinary Medicine, College of Animal Sciences, Zhejiang University, Hangzhou, Zhejiang China

**Keywords:** Porcine circovirus type 2, Capsid protein, Gold immunochromatographic assay, Polyclonal antibody

## Abstract

**Background:**

The existing detection methods for porcine circovirus type 2 (PCV2) specific antibodies in serum cannot determine the infection status, thus it is necessary to establish a method for detecting PCV2 antigen. The capsid protein (CAP) of PCV2, as a major structural protein that plays a significant role in viral replication and in inducing host’s immune response, is an ideal target antigen to monitor PCV2 infection. Therefore, a gold immunochromatographic assay (GICA) for rapid detection of PCV2 antigen based on the polyclonal antibodies (PAbs) against PCV2-CAP will be developed.

**Results:**

The truncated CAP protein (dCAP) was used to immunize rabbits to generate anti-serum. After preliminary purification by caprylic acid/ammonium sulfate precipitation (CAAS), specific PAbs were purified by affinity chromatography column coupled with dCAP and its titer was about two-fold higher than preliminary purified PAbs. Colloidal gold-PAbs conjugate was synthesized under the optimum conditions. The specific anti-dCAP PAbs and goat anti-rabbit antibody (GAR) were then sprayed onto nitrocellulose (NC) membrane as a test line (TL) and a control line (CL), respectively. The visual limit detection (vLOD) of the GICA strips was 5 ng/mL. Specificity assay indicated that the GICA strips had specifically detected PCV2 and was not reactive for porcine epidemic diarrhea virus (PEDV), pseudorabies virus (PRV), porcine reproductive and respiratory syndrome virus (PRRSV) or classic swine fever virus (CSFV). A total of 36 porcine serum samples were detected by this GICA and commercial enzyme-linked immunosorbent assay (ELISA) Kit, 9 positive samples were found by the developed strip with the rate of 25.0% comparing with 11 positive samples detected by the commercially ELISA Kit which positive rate was 30.5%, and the receiver operating characteristic (ROC) curve revealed that the relative sensitivity and specificity of this GICA strip were 72.7 and 96.0%, respectively, with an area of 87.2%.

**Conclusions:**

This study established an efficient detection method with high sensitivity and specificity for the clinical diagnosis of PCV2 antigen, that will facilitate a rapid and convenient way to evaluate the infection status of vaccinated pigs.

**Supplementary Information:**

The online version contains supplementary material available at 10.1186/s12917-022-03471-6.

## Introduction

Porcine circovirus type 2 (PCV2) is a member of genus circovirus, the smallest non-enveloped, single-stranded, circular DNA viruses that replicate autonomously in mammalian cells [1] and is the causative agent of the porcine multi-system wasting syndrome (PMWS), as characterized by wasting and jaundice in weaned piglets [2]. It was first discovered in Canada in 1991, and subsequently discovered in other countries such as the United States, France, and Japan [3]. In addition, PCV2 infection has been linked with porcine dermatitis nephrotic syndrome (PDNS), porcine respiratory disease syndrome (PRDC), reproductive disorders, granulomatous enteritis, necrotizing lymphadenitis, exudative epidermitis, and congenital tremor [4]. Recently, PMWS has become a severe problem affecting the Chinese pig industry [5–8], thus a rapid and sensitive detection method for clinical use is in urgent need.

The genome of PCV2 is a single-stranded negative-stranded circular DNA consisting of 1767 to 1768 nucleotides, and two major open reading frames (ORFs) [9]. ORF1 encodes two replication-related proteins (Rep and Rep’), and ORF2 encodes the viral capsid protein (CAP), which has good immunogenicity [10]. It was reported that PCV2 anti-serum can recognize the truncated CAP protein (dCAP), and the dominant epitope is located at 97 to 141 amino acids [11]. These characteristics make CAP protein an ideal target for developing detection methods. Serological diagnosis, such as Enzyme-linked immunosorbent assay (ELISA) [12], immunohistochemistry assay (IHC) [13], indirect immunofluorescence (IFA) [14], and immunoperoxidase monolayer cell assay (IPMA) [15] are the most common diagnostic methods for detecting PCV2 antibodies. But ELISA, IPMA, IHC or IFA have the disadvantages of requiring qualified personnel or high costs for the reagents and equipment. On the other hand, gold immunochromatographic assay (GICA) strips are rapid, simple, and low-cost, and can be used for a large number of clinical samples by any untrained individual.

It is well known that PCV2 vaccine can effectively improve production performance, but cannot eliminate the virus in pigs [16]. Performing the antibody monitoring alone cannot reflect the viral load in pigs, but when combined with antigen detection, we can more accurately evaluate the infection or vaccination situation. In this study, we used Cyanogen bromide (CNBr) dCAP affinity chromatography column to purify specific rabbit anti-PCV2-dCAP polyclonal antibody, and developed a GICA strip for PCV2 antigen detection, which lays the foundation for more rapid and accurate detection of PCV2 and helps us to analyze the infection status in vaccinated pigs better.

## Results

### Production of PAbs against PCV2-dCAP

**Fig. 1:**
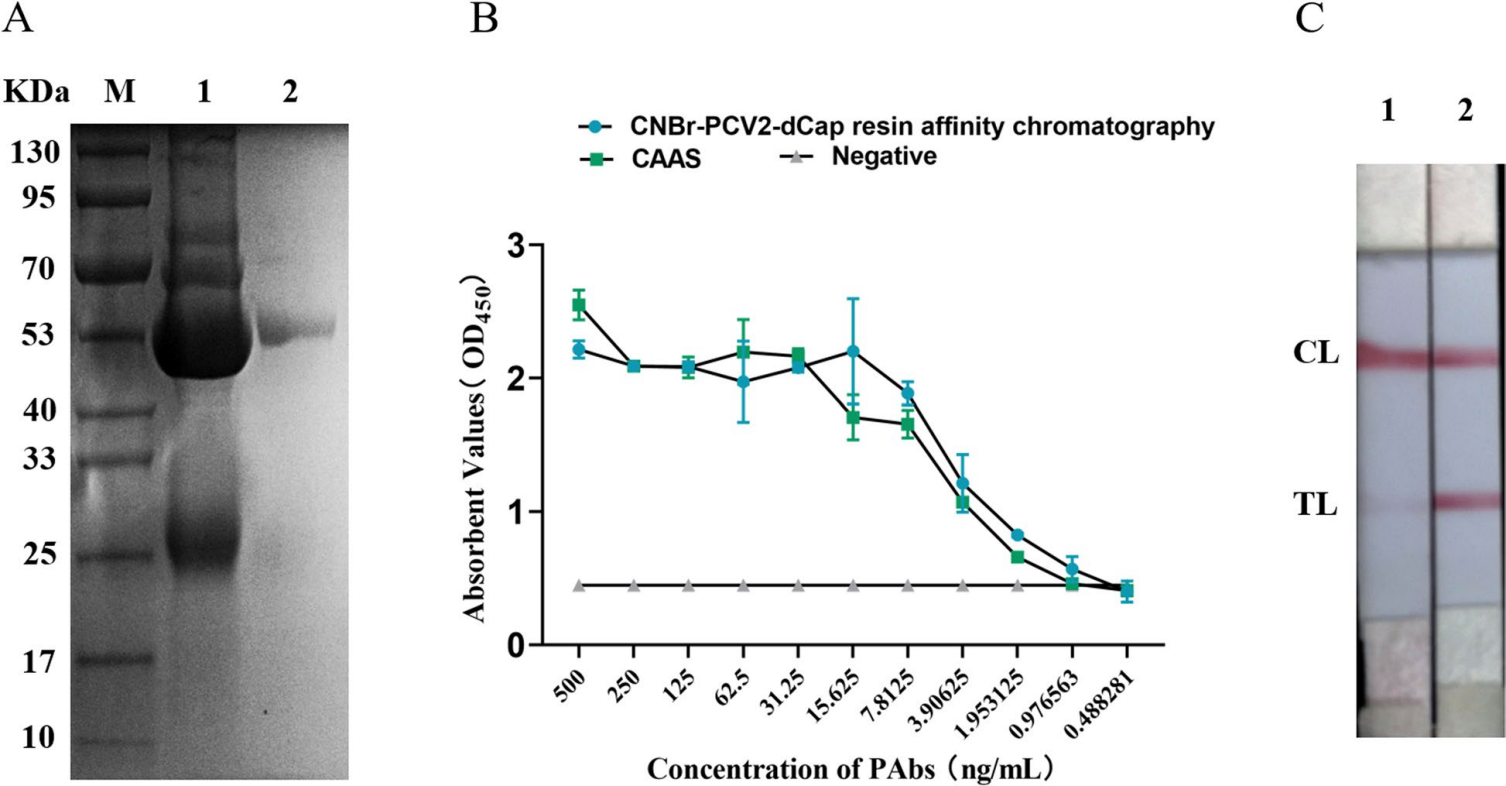
Purification and evaluation of anti-dCAP PAbs. **A** polyclonal anti-serum was purified by CAAS and then analyzed by SDS-PAGE. M represents a protein marker range from 10 to 130 KDa; 1 and 2 represent PAbs purified by CAAS and CNBr-PCV2 dCAP resin affinity chromatography, respectively; **B** The titers of PAbs were detected by indirect ELISA; **C** 1 and 2 represent the GICA strips was developed based on PAbs purified by CAAS and CNBr-PCV2 dCAP resin affinity chromatography; CL represents control line; TL represents test line

Anti-serum was preliminarily purified by CAAS method and further purified by the CNBr-PCV2-dCap resin affinity chromatography. The purity of PAbs purified by CAAS and affinity chromatography were both evaluated by polyacrylamide gel electrophoresis (SDS-PAGE), and the detection effects were evaluated by GICA. The result of SDS-PAGE revealed that the purity of PAbs purified by affinity chromatography was better than that of PAbs purified by CAAS (Fig. 1A). Besides, the antibody titer of PAbs purified by affinity chromatography reached 1:2048000 and was about 200% higher than the PAbs purified by CAAS (Fig. 1B). The result of GICA illustrated that the PAbs purified by affinity chromatography could recognize the dCAP protein better (Fig. 1C).

### Optimal pH and concentration of the conjugate PAbs

**Fig. 2:**
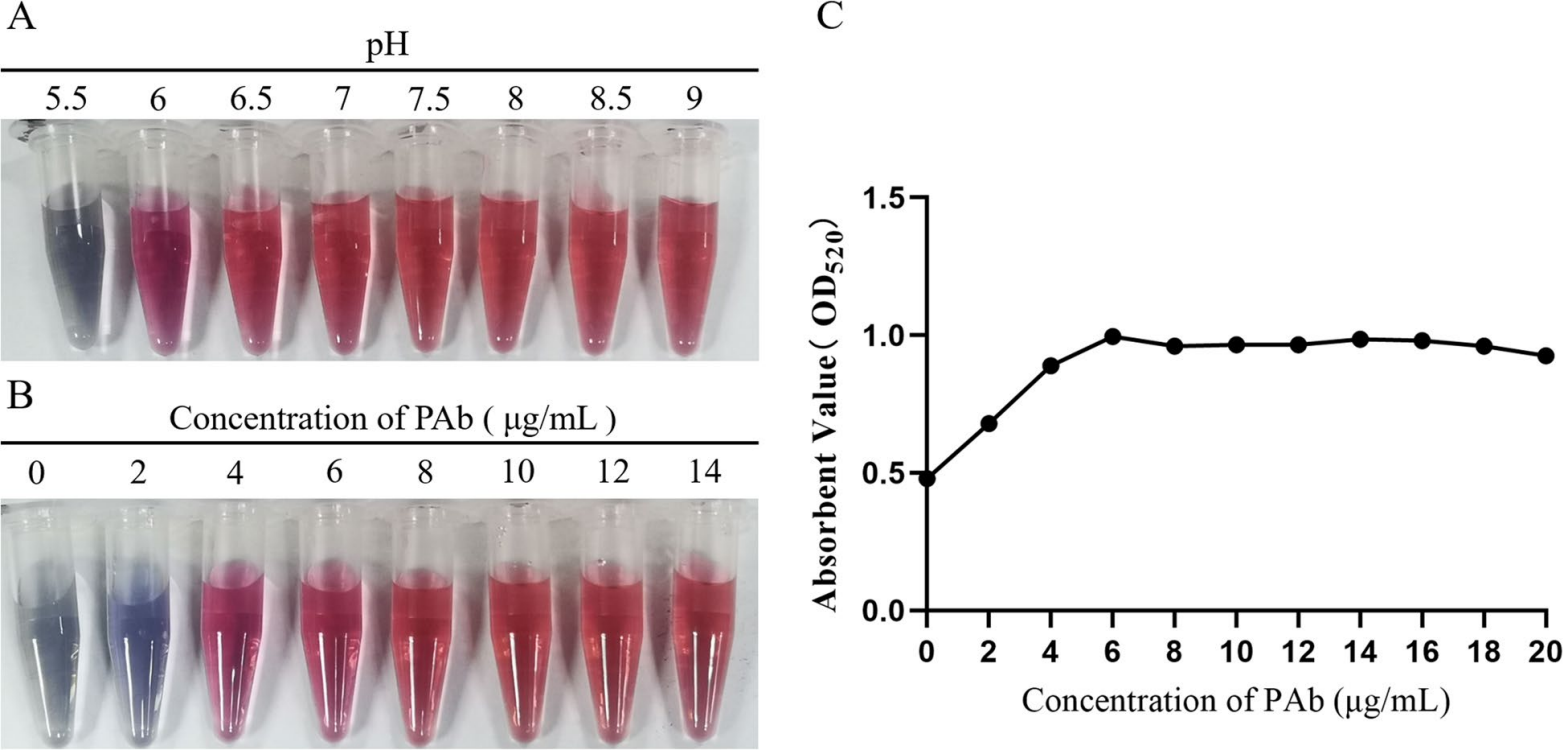
The optimum pH and conjugate concentration of PAbs. **A** Colloidal gold solution was adjusted to different pH values by 0.2 M/L Potassium carbonate to obtain optimum pH value; **B** and **C** A two-fold series increasing amount of PAbs were added into colloidal gold solution to obtain optimum conjugate amount of PAbs by directly observing or determining with UV spectrophotometer

The pH of PAbs for conjugation with the colloidal gold solution of red invariant was about 8 (Fig. 2A), and the minimum PAbs concentration labeled with colloidal gold of maximum absorbent value was 6 μg/mL, which was consistent with the observed result of red invariant. Therefore, the optimum concentration was 8 μg/mL according to the description in the method (Fig. 2B and C).

### Sensitivity, specificity, and stability of the GICA strips

**Fig. 3:**
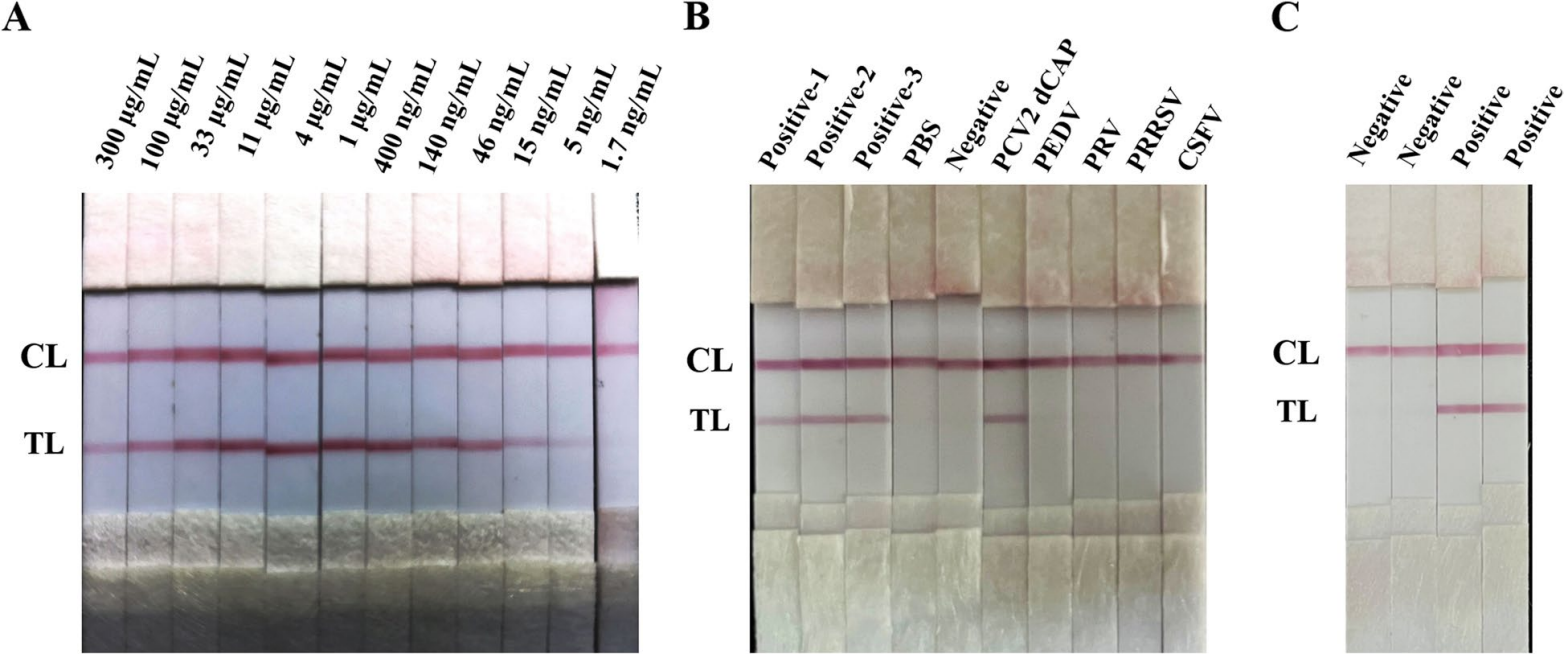
Sensitive, specificity and stability of the GICA strips. CL represents control line; TL represents test line; **A** The detection limited of the GICA strips was determined by detecting a three-fold dilution of about 0.8 mg/mL of recombinant PCV2 dCAP proteins. **B** The specificity of the GICA strips was determined by detecting PCV2 positive porcine samples (positive-1, positive-2 and positive-3) and negative samples, PCV2-dCAP protein, positive porcine serum for Porcine epidemic diarrhea virus (PEDV), pseudorabies virus (PRV), porcine reproductive and respiratory syndrome virus (PRRSV) and classic swine fever virus (CSFV). PBS was detected as a blank control. **C** The GICA strips developed was stored in 4 °C to determine the stability by detecting PCV2 positive and negative porcine samples

The sensitivity of the GICA was detected by three-fold serial dilutions of recombinant dCAP protein ranged from 300 μg/mL to 1.7 ng/mL, and the results indicated that the visual limit detection (vLOD) of the GICA strips was 5 ng/mL (Fig. 3A).

Three PCV2 positive porcine serum samples, dCAP protein and other porcine serum samples positive with PEDV, PRV, PRRSV and CSFV were used to evaluated the specificity of the strip. The results showed that the GICA strips for detection of three PCV2 positive porcine serum samples and PCV2 dCAP protein presented TL and CL, and others were only presented CL (Fig. 3B).

The GICA strips were stored at 4 °C for 24 weeks, and examined by PCV2 positive and negative porcine serum samples to evaluate the stability. The results demonstrated that the GICA strips stored at 4 °C for 24 weeks and detected the PCV2 positive porcine serum sample that still presented the CL and TL (Fig. 3C).

### Clinical test of the GICA strips

**Fig. 4:**
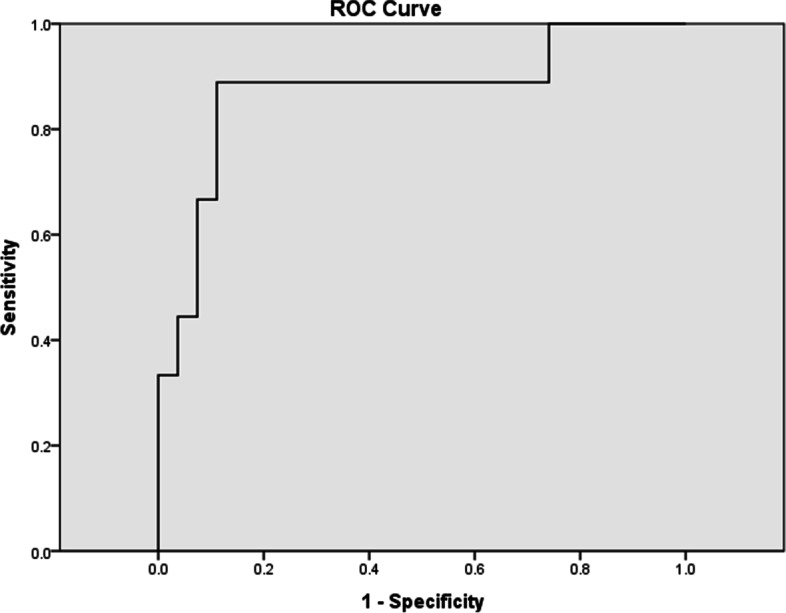
ROC curve analysis between the GICA strips and ELSIA. The area under of the ROC curve (AUC) was 0.872

Thirty-six of porcine serum samples were detected by the developed strips and commercial ELISA Kit. Nine samples and 27 samples were detected as positive and negative by the GICA strips, respectively (Fig. S1). Besides, 11 samples and 25 samples were detected as positive and negative by the commercial ELISA Kit, respectively (Table S1). Comparing to 30.0% positive rate of commercial ELISA Kit, the positive rate of the developed GICA strips was 25.0%. The agreement rate was 88.9% when the commercial ELISA Kit was treated as the standard method (Table 1). The ROC curves demonstrated that the relative sensitivity and specificity were 72.7 and 96.0% with an area of 87.2% (95% confidence interval (CI) 0.6492 to 0.9063, *P* < < 0.0001) (Fig. 4).

**Table 1:** Test results of 36 serum samples by the GICA strips and commercial ELISA kit

		GICA strips		
		Positive	Negative	Total
**ELSIA Test**	**Positive**	**8**	**3**	**11**
	**Negative**	**1**	**24**	**25**
	**Total**	**9**	**27**	**36**

*Abbreviations*: *GICA* gold immunochromatographic assay, *ELISA* enzyme-linked immunosorbent assay

**Fig. 5:**
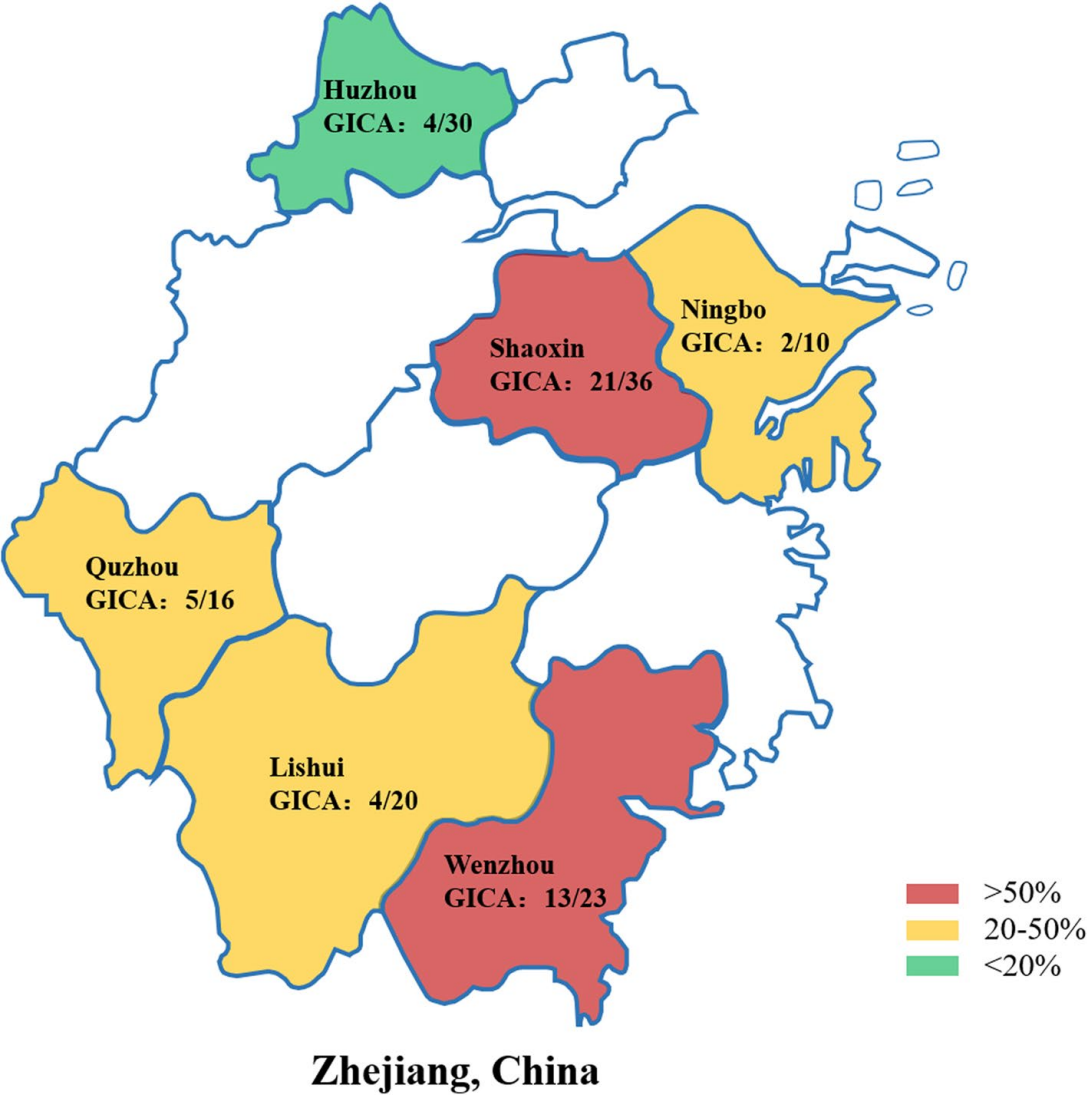
The positive rate of PCV2 in main pork production areas in Zhejiang province was detected by the GICA strips

A total of 135 serum samples from healthy pigs were detected to analyze the antigen seropositivity rate in different farms in Zhejiang, China. The results indicated that the antigen seropositivity rates of Huzhou, Quzhou, Lishui, Wenzhou, Shaoxin and Ningbo were 13.0, 31.3, 20.0, 56.5, 58.3 and 20.0%, respectively (Fig. 5).

**Fig. 6:**
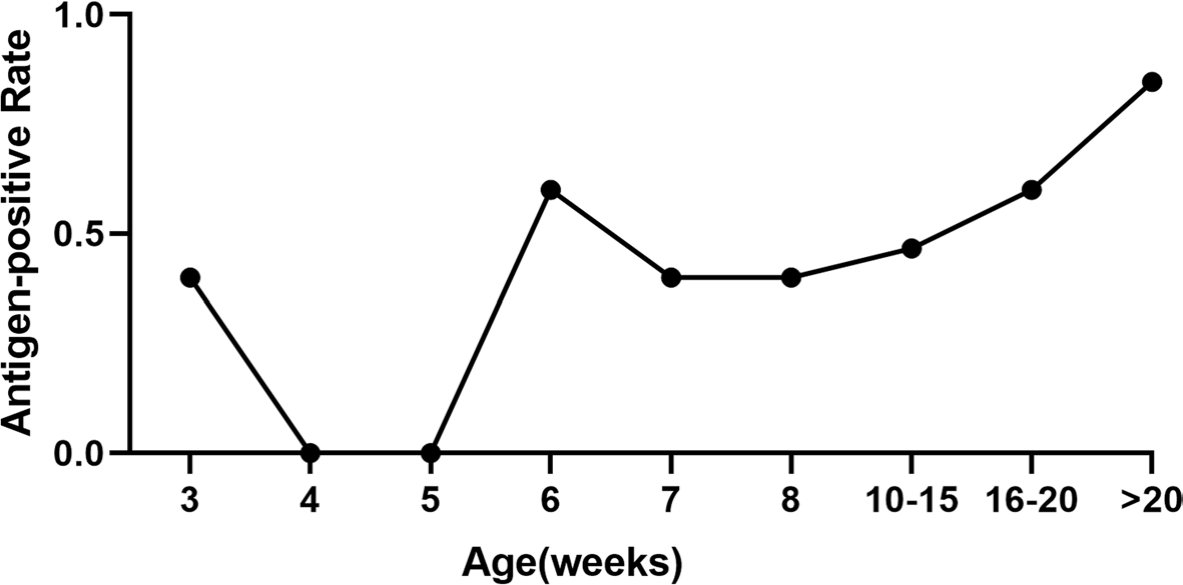
A total of 106 of PCV2 antibody-positive samples cover the whole life stage of porcine were also detected by the GICA strips

We also collected another 106 PCV2 antibody-positive samples including the whole life stage of porcine. Then the results of the detection illustrated that the PCV2 antibody-positive samples were not all antigen-positive, and the antigen-positive rate was increasing with increase in age. The antigen-positive rate of porcine age from 2 to 23 weeks was 66.2% (Fig. 6).

## Discussion

In recent years, the PCV2b genotype has been gradually replaced by the PCV2d genotype that emerges as the most prevalent in major pork producing areas, including the United States, China, Korea, and Vietnam [17–21]. Since the first imported vaccine being available in China at the end of 2009, there are more and more commercial vaccines available based on different PCV2 genotypes (PCV2a, PCV2b, and PCV2d). However, it also has been reported that the PCV2 vaccine based on genotype PCV2b was more efficient in boosting immunity against PCV2b than other genotypes [22]. Therefore, the current vaccines have been efficient in inducing humoral immune response and improving the production performance, but cannot abolish PCV2-infection. The commercial PCV2 detection Kits were commonly used to detect the antibody titers in serum to evaluate the effects of the vaccines. Since most pigs are vaccinated against PCV2 and PCV2 has been increasing in prevalence in China, it is difficult to know the PCV2 infection status of vaccinated pigs [23]. To estimate PCV2 antigen as a measure of the viral load in body fluids can help us know the effects of vaccines better. Therefore, we developed a method based on GICA to detect PCV2 antigen in serum.

In our previous study, we removed the N-terminal 41 amino acids of the PCV2 ORF2(GenBank: MG245866.1), and a large amount of this truncated protein was expressed in *Escherichia coli* (*E. coli*), and named as PCV2-dCAP, which was used as an immunogen and injected into rabbits to produce the PAbs [24]. The use of PAbs over monoclonal antibodies (MAbs) has its own advantages. PAbs are heterogeneous and produced from a large number of B cell clones, which allows them to bind to a wide range of antigen epitopes [25]. In contrast to MAbs’ single epitope, the effect of change on a single or small number of epitopes of PCV2 ORF2 is less likely to be significant for PAbs to recognize various PCV2 genotypes. Liu et al. collected a large number of porcine samples in Fujian, China and performed the amino acid sequence analysis of the isolated PCV2 ORF2, which indicated that there are many different PCV2 genotypes existing in China [26]. Therefore, the method based on PAbs may more likely to detect different PCV2 genotypes from various areas.

And then, the anti-serum was purified by CAAS. A total of 80 μg of purified PAbs were then mixed with 10 mL of gold nanoparticles with a mean diameter of 20 nm at pH 8. The above colloidal gold-PAbs conjugates were carried out to perform GICA. The result showed that the GICA strips presented a brightly red CL and an indistinct TL (Fig. 1C). The concentration of specific antibody in polyclonal sera is 50 to 200 μg/mL, and the range of total IgG concentration in sera is between 5 and 20 mg/mL [25]. So, the PAbs purified by CAAS were not suitable to construct the GICA strips that recognized PCV2 antigens.

PCV2-dCAP specific affinity·purification was carried out to improve the purity and specificity of polyclonal antibody. After PCV2-dCAP specific affinity·purification, 0.4 mg of specific anti-dCAP PAbs were obtained from 10 mg of PAbs. So, the obtained anti-dCAP PAbs were more specific, and could recognize a wide range of PCV2-dCAP epitopes. The results illustrated that the titer of the specific anti-dCAP PAbs was improved and the GICA strips based on the specific anti-dCAP PAbs presented a brightly red CL and TL (Fig. 1B and C).

Recent study has reported that the detection limit of their GICA strips based on MAbs was low to 2.16 ng/mL [27]. The detection limit of GICA strips in our study that was based on the specific anti-dCAP PAbs also could reach 5 ng/mL (Fig. 3A). The concentration and purity levels of specific antibody are higher in MAbs, which may suitable for the development of the GICA strips. However, the GICA strips based on PAbs purified by specific affinity purification also displayed good detection performance. The GICA strips developed could be used for detection at least 24 weeks after storge at 4 °C, indicating excellent stability. Positive porcine serum samples for PCV2, PEDV, PRV, PRRSV and CSFV were used for specificity detection. Only positive porcine serum samples infected with PCV2 and dCAP protein can be detected by the GICA strips, showing no cross-reactivity with other positive samples (Fig. 3B). The relative agreement rate of the GICA strips was 88.9% (Table 1) and the area under ROC curve (AUC) was 0.872 (Fig. 4) and 95% confidence interval (CI) was 0.6492 to 0.9063. Eight positive results were detected using the GICA strips among 11 positive samples, only a few of them were detected as negative. Overall, the GICA strip is a useful tool to evaluate the immune status of vaccinated pigs which has provided reliable detection results. Moreover, the GICA strip method is capable of differentiating between vaccinated and infected animals. For instance, both PCV2 vaccination and PCV2 infection, elicit high levels of neutralizing antibody titers in serum, but PCV2 vaccination has shown effectiveness in reducing viral loads [28]. Thus, the detection of viral load using the GICA strip method through detecting the PCV2 antigen in serum could serve as ideal index to distinguish between vaccination and infection.

Another 139 samples from different pig-farm in Zhejiang, China were detected by this strip. The infection rates of most detected farms were 20.0–50.0% or larger than 50.0%, and one farm was lower than 20.0% (Fig. 5). Huang et al. collected clinical samples from different provinces in China and founded that the positive rate of PCV2 was 53.0% [29]. The PCV2 infection rate in Zhejiang are lower than that in the whole of China. The results might illustrate that the generally vaccinated in Zhejiang province played a significant role in taking control of the PCV2 infection.

The detection of 106 PCV2 antibody-positive samples indicated that the antigen-positive rate increased from 5 weeks piglets to lager than 20 weeks pigs. The GICA strips cannot detect the antigen in the piglets (4–5 weeks) serum (Fig. 6). Woźniak et al. performed the quantitative RT-PCR to detect the PCV2 DNA in piglets, weaners and fatteners, and it showed that PCV2 DNA was more frequently found in weaners (5–8 weeks old) and fatteners (> 9 weeks old) [30]. The results of DNA detection were similar with our results of GICA strips against PCV2 antigen. Previous studies revealed that the PCV2 maternal antibody half-life in piglets is 3 weeks, so the maternal antibody may give the piglets a protection to clear the PCV2 [31].

The GICA is a method to detect the antigen in serum, it is different from the quantitative PCR method that detect the DNA amount in sample. The GICA strip could be used as a qualitative method to evaluate the infection status, but not a quantitative method to account for the accurate viral loads [32]. So, In the further study, we aim to develop the method based on GICA which capable to evaluate the viral load in quantitative level.

## Conclusion

In conclusion, a GICA strip based on polyclonal antibody against PCV2-dCAP was established for the detection of porcine circovirus antigen, and this strip was good in sensitivity, specificity, and accuracy. In the context of widespread PCV2 infection and vaccination, only performing the detection for antibody couldn’t evaluate effects of vaccines. Therefore, this GICA strip based detecting antigen is a valid and rapid method to let us know the PCV2 infection status of vaccinated pigs, so it can help us to evaluate the vaccines better.

## Methods

### Animals and samples

Rabbits were purchased from Zhejiang provincial laboratory animal center. PCV2 dCAP protein was prepared and stored in our laboratory; positive serum for Porcine epidemic diarrhea virus (PEDV), pseudorabies virus (PRV), porcine reproductive and respiratory syndrome virus (PRRSV) and classic swine fever virus (CSFV) and 36 clinical porcine serum samples and 106 antibody positive serum samples for PCV2 were donated by the College of Animal Science of Zhejiang University and Zhejiang Academy of Agricultural Sciences. A total of 135 serum samples from healthy pigs were collected from different farms in Zhejiang province, China and also donated by Zhejiang Academy of Agricultural Sciences.

### Production of polyclonal anti-serum

Polyclonal anti-serum against dCAP were generated in rabbits by immunizing it with recombinant dCAP protein. Briefly, two healthy rabbits were immunized with a mixture containing 200 μg of dCAP protein and Freund’s adjuvant at the ratio of 1:1. The whole immunization procedure contained three injections. Each injection was carried out at an interval of 2 weeks. Serum was collected at 7 days after final immunization and antibody titer of three times’ serum was determined by indirect ELISA.

### Anti-serum against dCAP was purified by caprylic acid/ammonium sulfate precipitation (CAAS)

Rabbit anti-serum against PCV2 dCAP protein was purified by CAAS method, as previously described [33]. Briefly, each aliquot of anti-serum (5 mL) was diluted 3-fold with acetate buffer (hydrogen ion concentration (pH) 4.0) and adjusted to a pH at 4.5 with 0.1 mol/L Sodium hydroxide. Octanoic acid (170 μL) was added dropwise with constantly stirring. The precipitate containing non-IgG proteins were removed by centrifugation (10,000 g × 20 mins). The supernatant was adjusted to pH at 7.5 by dialyzed in 500 mL of 0.01 M phosphate-buffer saline (PBS) overnight at 4 °C. The IgG in 0.01 M PBS solution was then precipitated by the addition of Saturated Ammonium Sulfate (20 mL) under constantly stirring. The precipitate obtained was dissolved to the original volume with PBS (5 mL) and dialyzed by using 500 mL of 0.01 M PBS overnight at 4 °C.

### Preparation of specific PAbs against PCV2-dCAP by CNBr-PCV2-dCAP affinity chromatography

PCV2-dCAP protein (20 mg) was dialyzed in 500 mL of 0.1 M sodium carbonate (pH 8.3) overnight at 4 °C. CNBr-activated agaroses (Sangon, C500099, China) (1 mL) were packed in column and washed by 5 mL of 1 mM HCL. CNBr-activated agaroses were then mixed with PCV2 dCAP protein for 6 hours at 25 °C to obtain the CNBr-PCV2-dCAP agarose. Then the prepared agaroses were washed by 10 mL of 1 M Tris-HCl (pH 8.3) to block the residual agaroses, and 10 mL of 1 M sodium chloride was used to remove uncoupled proteins. The prepared agaroses were stored in 20.0% ethanol solution at 4 °C. The PAbs purified by CAAS were subsequently added to the column that had been packed with CNBr-PCV2-dCAP agaroses. The agaroses were then washed with PBS until that the absorbance of the flow-through faction at 280 nm approaches baseline. The specific PAbs were then eluted with 1 M Tris-HCl (pH 2.7), and each fraction was collected in separate tubes. Sodium dodecyl sulfate SDS-PAGE was performed to analyze the purity of the prepared specific PAbs. The specific PAbs were dialyzed against 500 mL of PBS at 4 °C.

### Determination of antibodies titers by indirect ELISA

The antibody titer of PAbs were determined by indirect ELISA according to a previously described method [34]. Briefly, the concentration of the PAbs purified by CAAS and the specific PAbs were determined by Bradford Protein Assay Kit (Sangon, C503031, China) and then diluted to 1 mg/mL. The wells of a 96-well microtiter plate was coated with 100 μL of 5 μg/mL recombinant PCV2 dCAP protein and incubated overnight at 4 °C. The unbound protein was discarded and the plate was washed three times with PBST. After blocking for 1 h, the plate was also washed three times with PBST and incubated with the PAbs diluted with PBST at 1:1000 for 2 h at 37 °C. And with three times washing, the plate was incubated with HRP-Goat anti-Rabbit polyclonal antibody (Affinity, S0001, China) diluted with PBST at 1:3000 for 2 h at 37 °C. Finally, the plate was washed for three times and incubated with 200 μL TMB (beyotime, P0209, China) for 10 to 30 min at 37 °C and added with 50 μL of stopping solution. The absorbance at 450 nm was measured with a microtiter plate reader.

### Synthesis of colloidal gold

A total volume of 500 mL of Colloidal gold nanoparticles with a mean diameter of 20 nm were produced by reduction of 5 mL of 1.0% Chloroauric acid (Sangon, A602523, China) with 0.1 g of 1.0% sodium citrate according to a previously described method [35]. Briefly, beakers and flasks were washed with ultrapure water for six times and dried before use. The ultrapure water (500 mL) was heated to boiling point, and then 5 mL of 1.0% Chloroauric acid solution were added quickly into the water under constantly stirring. A total 0.1 g of 1.0% sodium citrate was then added to the water. When the color of the solution changed from blue to bright red, the solution was heated for another 5 mins, and the cooled colloidal gold solution was stored at 4 °C.

### Preparation of colloidal gold-PAbs conjugate

The optimal pH of the PAbs for conjugation with the colloidal gold solution was firstly determined. Briefly, each 1 mL of colloidal gold solution was adjusted into different pH (5, 5.5, 6, 6.5, 7, 7.5, 8, 8.5, 9) by the addition of 0.2 M/L Potassium carbonate and mixed with 100 μL of PAb that had been diluted with PBS (0.01 M, pH 7.4) at the concentration of 100 μg/mL, with slight shaking for 10 mins. Then 100 μL of 10.0% sodium chloride solution was added into each 1 mL of colloidal gold solution and stirred for another 10 mins. After 1 hour incubation at room temperature, the color of the mixtures changed from blue to bright red as the pH increases, and the optimum pH of PAbs for colloidal gold conjugation was the minimum pH of red invariant.

The 1 mL of colloidal gold solution was adjusted to the optimum pH and added 100 μL of different concentration PAbs (0, 20, 40, 60, 80, 100, 120, 140, 160, 180, 200 μg/mL), with slight shaking for 10 mins. Then 100 μL of 10.0% sodium chloride solution was added into each 1 mL of colloidal gold solution and stirred for another 10 mins. The absorption at 520 nm was detected. The optimal concentration for PAbs labelled colloidal gold was 20.0% higher than the minimum concentration.

Ten mL of colloidal gold was adjusted to the optimum pH and mixed with the optimum concentration of PAbs to prepare the colloidal gold probe. After stirring at intervals for 30 mins, the mixture was added with 1 mL of 10.0% bovine serum albumin (BSA) solution to block the un-reacted sites of the gold nanoparticles, with stirring for another 1 hour, and then centrifuged at 12,000 rpm for 30 mins. After carefully removing the supernatant, the precipitate was re-suspended in 1 mL of dilution buffer (0.002 mol/L Tris buffer (pH 8.0) containing 20.0% sucrose, 5.0% trehalose and 1.0% BSA solution) and store at 4 °C for late use.

### Preparation of gold immunochromatographic strip

**Fig. 7:**
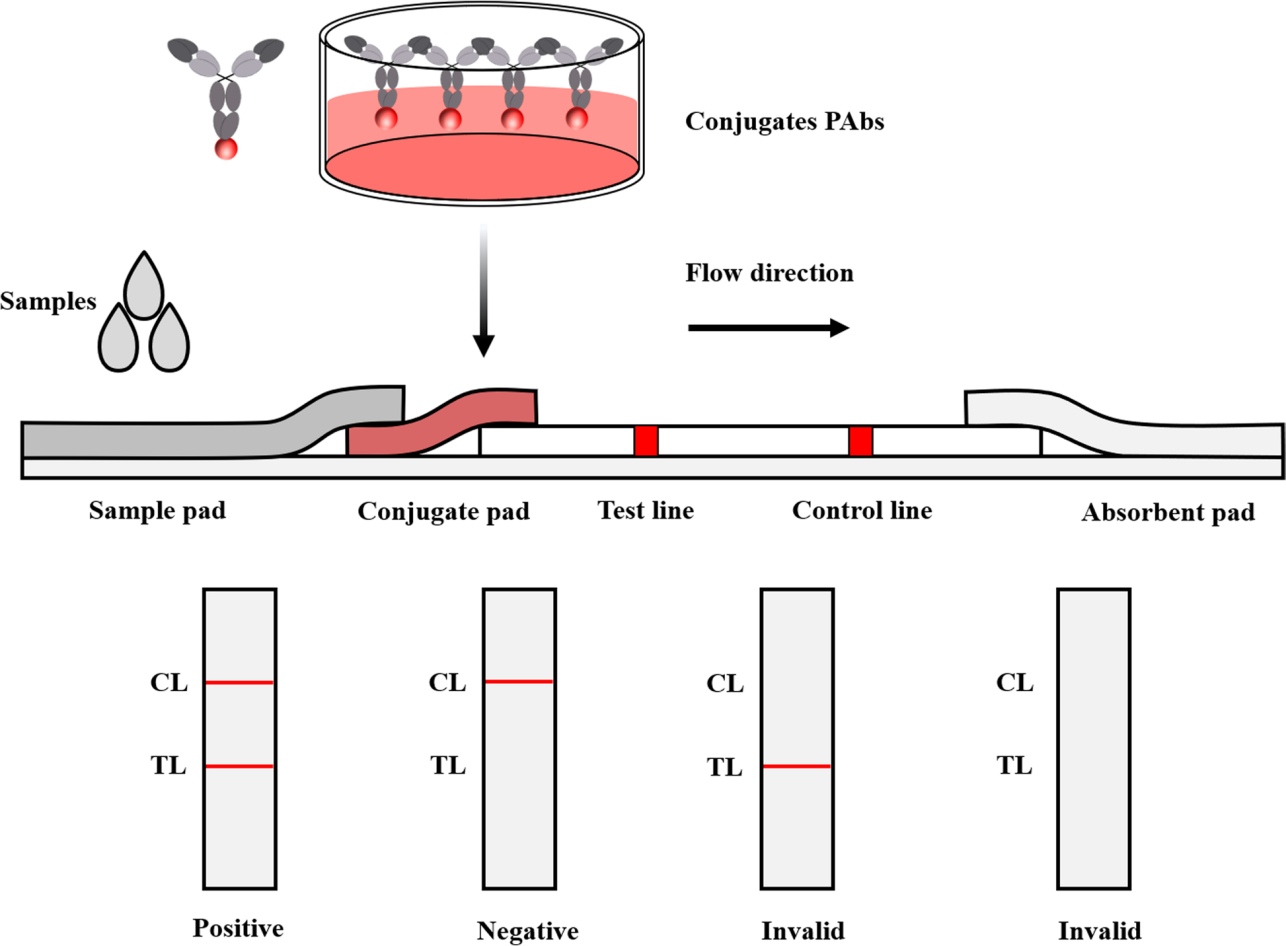
Structure of gold immunochromatographic assay strip, negative, positive and invalid schematic diagram. CL represents control line; TL represents test line

The glass fiber (Kinbio, RB65, China) was used as sample pad that had been saturated with 0.01 M borax (pH 7.4) containing 0.50% casein sodium, 1.0% polyvinyl pyrrolidone 100 and 1.0% Trition X-100. The specific PAbs were diluted into 1 mg/mL with 0.01 M PBS containing 3.0% trehalose. The diluted PAbs and goat anti-rabbit Mab (1 mg/mL) were transferred into the Nitrocellulose (NC) membrane (Sartorius, CN140, Germany) with a volume of 1 μL/cm to form the test line (TL) and control line (CL), respectively, with the Biodot XYZ3050™ Dispense System (BioDot, XYZ3050™, the United States). The colloidal gold probe was sprayed into the polyester fiber (Kinbio, DL98, China) as the conjugate pad. The GICA strip including the sample pad, conjugate pad, NC membrane and absorbent pad was described and assembled as in Fig. 7. The strips were then cut into 4 mm by the Programmable slitter (AUTOKUN, HGS201, China) and store at 4 °C for use. The GICA of two PAbs were carried out to compare their detection effects.

### Sensitivity, specificity, and stability of the GICA strips

The visual limit of the GICA strips was evaluated by different concentrations of recombinant dCAP protein. The recombinant dCAP protein was diluted into different concentrations (300 μg/mL, 100 μg/mL, 33 μg/mL, 11 μg/mL, 4 μg/mL, 1 μg/mL, 400 ng/mL, 140 ng/mL, 46 ng/mL, 15 ng/mL, 5 ng/mL, 1.7 ng/mL) to evaluate the limit of the GICA strips. Besides, 100 μL of 0.01 M PBS (pH 7.4) was used as the negative control.

The specificity of the GICA strips was examined by recombinant dCAP protein， porcine positive serum samples for PCV2 (positive-1, positive-2, positive-3), porcine epidemic diarrhea virus (PEDV), pseudorabies virus (PRV), porcine reproductive and respiratory syndrome virus (PRRSV) and classic swine fever virus (CSFV). PBS and PCV2 negative porcine serum sample were used as blank and negative control.

The GICA strips were stored at 4 °C to evaluate the stability with PCV2 positive serum samples after 24 weeks. PCV2 negative porcine serum sample was detected as negative control.

### Detection of clinical samples

A total of 36 porcine serum samples were detected by the GICA strips and Porcine(PCV-2 Ag)ELISA Kit (EK-Bioscience, EK-P41966, China) to evaluate the accuracy of the strips. Briefly, all Kit reagent from refrigerator should reach room temperature before starting the assay. Positive and negative control (50 μL) were added to the positive and negative wells respectively. One hundred μL of HRP-conjugate reagent was added to each well, and the plate was covered with an adhesive strip and incubated for 60 min at 37 °C. The reagent in each well was aspirated and washed for five times. After the last wash, the remaining wash solution in each well was removed. The chromogen solution A (50 μL) and chromogen solution B (50 μL) was added to each well, and the plate was mixed gently and incubated for 15 min at 37 °C. The stop solution (50 μL) was added to each well, and the optical density of the plate was read at 450 nm using a microtiter plate reader. One hundred thirty-five of serum samples from healthy pigs were collected from different pig farms in Zhejiang province and tested by the GICA strips. A total of 106 of PCV2 antibody-positive samples were also detected by the GICA strips.

### Statistical analyses

SPSS software version 20.0 was used to carry out statistical analysis and calculate the area under the curve (AUC) score of ROC curve. *P*-values < 0.05 were considered statistically significant. Graphs were made by GraphPad Prism 8.

## Supplementary Information

**Additional file 1: Supplement Fig. 1.** A total of 36 porcine samples were detected by the developed GICA strips.Number (1 to 36) represent 36 porcine samples.

**Additional file 2: Supplement Fig. 2.** The original figure of SDS PAGE of purified polyclonal antibodies.

**Additional file 3: Supplement Fig. 3.** Western blot analysis between recombinant antigen and purified polyclonal antibody. The blot membrane was cut prior to hybridization with polyclonal antibody A: the blot membrane was in the Brightfield; B: the blot membrane was imaged by the Bio-Rad ChemiDoc XRS+.

**Additional file 4: Supplementary Table 1.** A total 36 samples was detected by the commercial ELISA Kit and the OD values at 450 nm was presented in this table.

**Additional file 5: Supplementary Table 2.** The 135 porcine samples of different ages was detected by the GICA strips to evluation of positive rate in different ages of pigs.

## Data Availability

The authors confirm that the data supporting the findings of this study are available within the article [and/or] its supplementary materials.

## Abbreviations

PCV2: Porcine circovirus type 2
CAP: Capsid protein
GICA: Gold immunochromatographic assay
CAAS: Caprylic acid/ammonium sulfate precipitation
PAbs: Polyclonal antibodies
GAR: Goat anti-rabbit antibody
TL: Test line
CL: Control line
vLOD: Visual limit detection
PEDV: Porcine epidemic diarrhea virus
PRV: Pseudorabies virus
PRRSV: Porcine reproductive and respiratory syndrome virus
CSFV: Classical swine fever virus
ROC: Receiver operating characteristic
PMWS: Porcine multi-system wasting syndrome
PDNS: Porcine dermatitis nephrotic syndrome
PRDC: Porcine respiratory disease syndrome
ORFs: Open reading frames
Rep: Replication-related proteins
dCAP: Truncated CAP protein
ELISA: Enzyme-linked immunosorbent assay
IHC: Immunohistochemistry assay
IFA: Indirect immunofluorescence
IPMA: Immunoperoxidase monolayer assay
PBS: Phosphate-buffer saline
pH: Hydrogen ion concentration
SDS-PAGE: Sodium dodecyl sulfate polyacrylamide gel electrophoresis
BSA: Bovine serum albumin
MAbs: Monoclonal antibodies
*E. coli*: *Escherichia coli*
CNBr: Cyanogen bromide
