# Supplementary material for: Development of gold Immunochromatographic assay strip based on specific polyclonal antibodies against capsid protein for rapid detection of porcine circovirus 2 in Zhejiang province, China
Source: BMC Vet Res. 2022 Oct 18;18:373. doi: 10.1186/s12917-022-03471-6 (PMC9578217; doi:10.1186/s12917-022-03471-6)
Supplement: Supplementary file 1 — Additional file 1: Supplement Fig. 1. A total of 36 porcine samples were detected by the developed GICA strips.Number (1 to 36) represent 36 porcine samples. [file 12917_2022_3471_MOESM1_ESM.pdf]

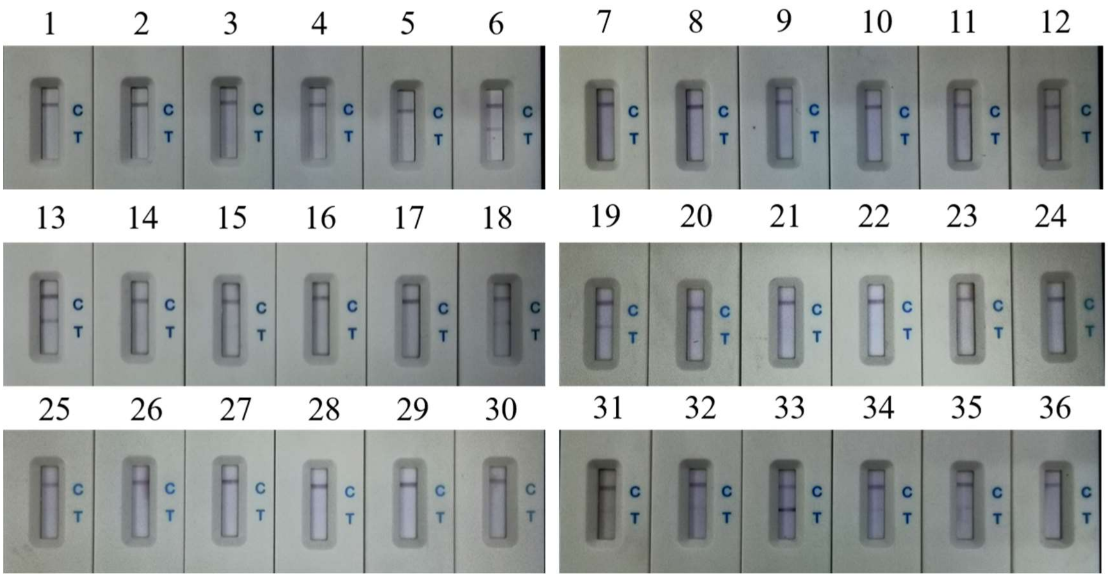

Supplement Fig. 1. A total of 36 porcine samples were detected by the developed GICA strips. Number (1 to 36) represent 36 porcine samples;
