# Supplementary figures and images for: Development of gold Immunochromatographic assay strip based on specific polyclonal antibodies against capsid protein for rapid detection of porcine circovirus 2 in Zhejiang province, China
Source: BMC Vet Res. 2022 Oct 18;18:373. doi: 10.1186/s12917-022-03471-6 (PMC9578217; doi:10.1186/s12917-022-03471-6)

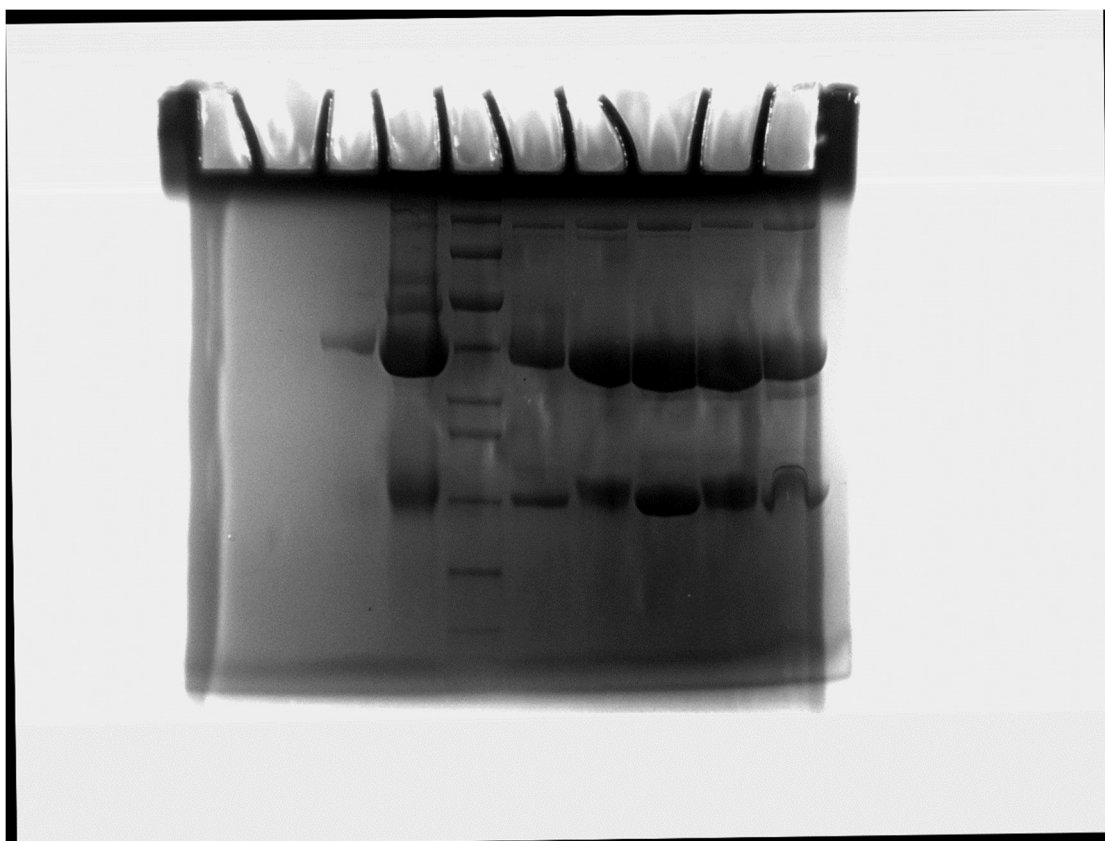

**Supplement Fig. 2. The original figure of SDS PAGE of purified polyclonal antibodies.**

Supplement: Supplementary file 2 — Additional file 2: Supplement Fig. 2. The original figure of SDS PAGE of purified polyclonal antibodies. [file 12917_2022_3471_MOESM2_ESM.pdf]
