# Supplementary material for: Development of gold Immunochromatographic assay strip based on specific polyclonal antibodies against capsid protein for rapid detection of porcine circovirus 2 in Zhejiang province, China
Source: BMC Vet Res. 2022 Oct 18;18:373. doi: 10.1186/s12917-022-03471-6 (PMC9578217; doi:10.1186/s12917-022-03471-6)
Supplement: Supplementary file 3 — Additional file 3: Supplement Fig. 3. Western blot analysis between recombinant antigen and purified polyclonal antibody. The blot membrane was cut prior to hybridization with polyclonal antibody A: the blot membrane was in the Brightfield; B: the blot membrane was imaged by the Bio-Rad ChemiDoc XRS+. [file 12917_2022_3471_MOESM3_ESM.pdf]

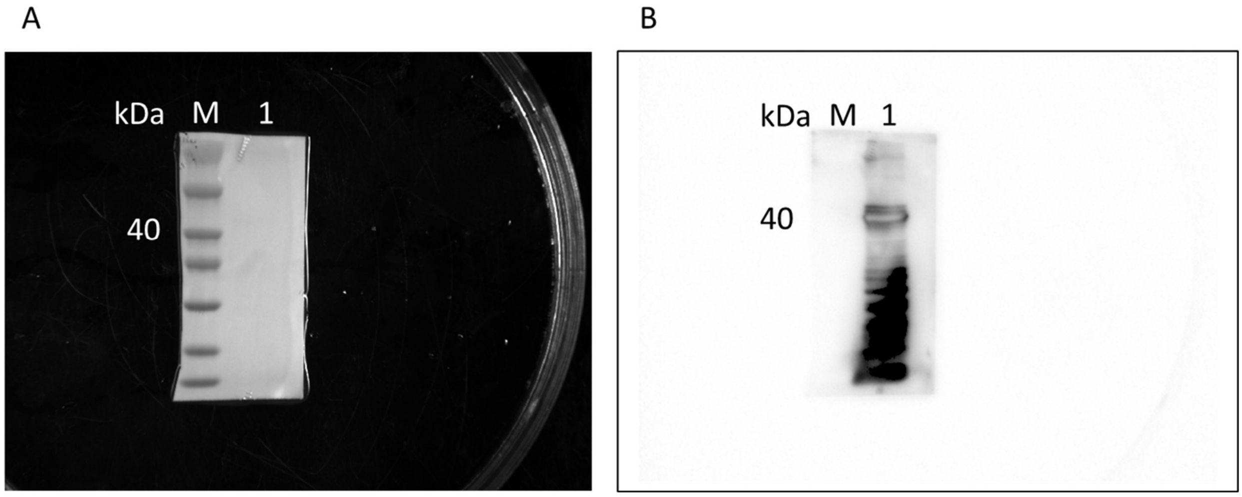

Supplement Fig. 3. Western blot analysis between recombinant antigen and purified polyclonal antibody. the blot membrane was cut prior to hybridization with polyclonal antibody A： the blot membrane was in the Brightfield; B: the blot membrane was imaged by the Bio-Rad ChemiDoc XRS+.
