# Supplementary material for: Development of gold Immunochromatographic assay strip based on specific polyclonal antibodies against capsid protein for rapid detection of porcine circovirus 2 in Zhejiang province, China
Source: BMC Vet Res. 2022 Oct 18;18:373. doi: 10.1186/s12917-022-03471-6 (PMC9578217; doi:10.1186/s12917-022-03471-6)
Supplement: Supplementary file 4 — Additional file 4: Supplementary Table 1. A total 36 samples was detected by the commercial ELISA Kit and the OD values at 450 nm was presented in this table. [file 12917_2022_3471_MOESM4_ESM.pdf]

Supplementary Table 1. A total 36 samples was detected by the commercial ELISA Kit and the OD values at 450 nm was presented in this table.

Sample Number	ELISA (OD450)
1	0.478
2	0.148
3	0.149
4	0.143
5	0.845
6	0.682
7	0.159
8	0.161
9	0.163
10	0.122
11	0.123
12	0.11
13	0.139
14	0.193
15	0.108
16	0.122
17	0.12
18	0.864
19	1.124
20	0.175
21	0.147
22	0.155
23	0.155
24	0.153
25	0.159
26	0.154
27	0.17
28	0.234
29	1.073
30	0.23
31	0.418
32	0.48
33	0.472
34	3.082
35	3.124
36	0.129
