# Supplementary material for: Development of gold Immunochromatographic assay strip based on specific polyclonal antibodies against capsid protein for rapid detection of porcine circovirus 2 in Zhejiang province, China
Source: BMC Vet Res. 2022 Oct 18;18:373. doi: 10.1186/s12917-022-03471-6 (PMC9578217; doi:10.1186/s12917-022-03471-6)
Supplement: Supplementary file 5 — Additional file 5: Supplementary Table 2. The 135 porcine samples of different ages was detected by the GICA strips to evluation of positive rate in different ages of pigs. [file 12917_2022_3471_MOESM5_ESM.pdf]

Supplementary Table 2. The 135 porcine samples of different ages was detected by the GICA strips to evluation of positive rate in different ages of pigs.

Age of Pigs （weeks）	Positive numbers	Total numbers	Positive rate
3	4	10	0.4
4	0	5	0
5	0	5	0
6	3	5	0.6
7	2	5	0.4
8	4	10	0.4
10-15	7	15	0.466667
16-20	6	10	0.6
>20	33	39	0.846154
